# Special Issue “Recent Advances in the Synthesis, Functionalization and Applications of Pyrazole-Type Compounds II”

**DOI:** 10.3390/molecules28155873

**Published:** 2023-08-04

**Authors:** Vera L. M. Silva, Artur M. S. Silva

**Affiliations:** LAQV-REQUIMTE, Department of Chemistry, University of Aveiro, 3810-193 Aveiro, Portugal

Pyrazole and its derivatives are considered privileged *N*-heterocycles with immense therapeutic potential. In current medicinal chemistry, the incorporation of a pyrazole nucleus is a common practice by which to develop new drug-like molecules with different bioactivities, such as anti-cancer, anti-diabetic, anti-inflammatory, anti-neurodegenerative, anti-bacterial, among others, giving rise to a great number of approved therapeutics. Pyrazoles are also found within a variety of agrochemicals (fungicides, insecticides, and herbicides) and are versatile scaffolds for synthetic manipulations. The structural features of pyrazoles—mainly tautomerism, with possible implications for their reactivity, their dyeing and fluorescence properties, as well as their diverse applications—have stimulated the work of several research groups towards the synthesis and functionalization of pyrazole-type compounds and the study of their properties. 

After the success of the first edition of this Special Issue, this second edition has the same goal—that is, to provide a broad survey of the most recent advances in pyrazole’s chemistry. 

In this second edition, ten original research articles and five reviews covering some of the most recent advances in the synthesis, transformation, properties, and relevant applications of pyrazoles are reported. A. Žukauskaitė, E. Arbačiauskienė and their coworkers reported a regioselective strategy for synthetizing ethyl 1-(oxiran-2-ylmethyl)-3-aryl-1*H*-pyrazole-5-carboxylates from easily accessible 3(5)-aryl-1*H*-pyrazole-5(3)-carboxylates. By using different bases, reaction media, and temperatures, regioselective alkylation was achieved, and the established conditions were applied to the synthesis of novel pyrazolo[1,5-*a*][1,4]diazepin-4-ones via the ring-opening of the oxirane with amines and a direct cyclisation sequence. They further applied this synthetic strategy to investigate the reactivity of ethyl 1*H*-indole-2-carboxylate and ethyl benzo[*d*]imidazole-2-carboxylate scaffolds, which led to the formation of additional fused tetrahydro[1,4]diazepino[1,2-*a*]indol-1-one and tetrahydro-1*H*-benzo[4,5]imidazo[1,2-*a*][1,4]diazepin-1-one derivatives [1]. Polyfunctionalized pyrazoles, of general interest in medicine and material sciences, were the subject studied by M. Jasiński and coworkers, who described a solvent-free two-step mechanochemical synthesis of trifluoromethylated and non-fluorinated polysubstituted pyrazoles in moderate-to-high regioselectivity and fair yields, starting from simple substrates, chalcones, and hydrazonoyl halides. The protocol comprises [3 + 2]-cycloaddition of in situ-generated nitrile imines and chalcones, followed by the oxidation of the initially formed 5-acylpyrazolines with activated MnO_2_. The second step proceeds via an exclusive deacylative pathway to give a series of 1,4-diarylpyrazoles functionalized with a fluorinated (CF_3_) or non-fluorinated (Ph, COOEt, Ac) substituent at C-3 of the heterocyclic ring. They have also shown that, in contrast, the MnO_2_-mediated oxidation of a model isomeric 4-acylpyrazoline proceeded with low chemoselectivity, leading to fully substituted pyrazole as a major product formed via dehydrogenative aromatization. This work also evidenced the application of nitrile imines as building blocks for organic synthesis [2]. R. Jasiński and coworkers used molecular electron density theory (MEDT) to explain the unexpected formation of 1-(4-bromophenyl)-3-phenyl-5-nitropyrazole in the reaction between (*E*)-3,3,3-trichloro-1-nitroprop-1-ene and *N*-(4-bromophenyl)-C-arylnitrylimine. Although the theoretical MEDT studies showed that both possible [3 + 2]-cycloaddition pathways may occur from a kinetic point of view, of which the formation of 1-(4-bromophenyl)-3-aryl-4-trichloromethyl-5-nitro-Δ^2^-pyrazoline was more probable, the experimental results showed that this reaction occurred with full regioselectivity. However, the extremely unstable obtained products were spontaneously converted through a unique CHCl_3_-elimination process to give the unexpected pyrazole systems [3]. A. Spallarossa and coworkers reported the regio- and chemoselective one-pot synthesis of a small library of highly functionalized phenylaminopyrazoles by the one-pot condensation of active methylene reagents, phenylisothiocyanate, and substituted hydrazine. Preliminary cell-based assays performed via the MTT method on a panel of eight tumor cell lines (namely, breast cancer: MCF7, MDA-MB231, SK-Br3; melanoma: SKMEL-28; ovarian cancer: SKOV-3; liver cancer: Hep-G2; cervical cancer: HeLa; and lung cancer: A549) and one normal human fibroblasts cell line (GM-6114) showed that these phenylaminopyrazoles are poorly cytotoxic against both the selected cell lines [4]. L. Kayukova, A. Vologzhanina, and their coworkers demonstrated that the reaction products of the nitrobenzenesulfochlorination of β-aminopropioamidoximes strongly depend on the structure of the initial substrates and temperature. In addition, the authors reported the results of the in vitro screening of the library of nitrobenzenesulfochlorination products for anti-diabetic activity. Two distinct compounds, obtained in the *p*-nitrobenzenesulfochlorination of β-(thiomorpholin-1-yl)- and β-(benzimidazol-1-yl)propioamidoximes showed high α-glucosidase activity superior to the activity of the acarbose standard [5]. J.-C. Rodríguez-Ubis and coworkers reported an orthogonal synthetic approach to non-symmetrical bisazolyl 2,4,6-trisubstituted pyridines, important ligands in a variety of transition and lanthanide ions complexes, starting from the readily available 4-bromo-2,6-difluoropyridine. Both fluorine atoms allow for easy selective stepwise substitution, and the bromine atom provides easy access to additional functionalities through both Suzuki and Sonogashira Pd(0) cross-coupling reactions. This strategy gives access to pyridines with different substituents on the pyrazole, indazole, and pyridine heterocycles, it being possible to tune the electronic π character of these trisheterocyclic units to obtain the best candidates for ligands [6]. On the basis of the formation of four-coordinate boron(III) complexes in the reaction of 1-(2-pyridinyl)pyrazol-5-one derivatives with arylboronic acids in basic media, K.-I. Lee and coworkers revealed the key structural requirements of 1-(2-pyridinyl)pyrazol-5-ones for disproportionation of boronic acids, which are the use of unprotected acid and the presence of a [*N*,*O*]-bidentate ligand. Although the exact mechanism of this disproportionation is not clear, the reported method is particularly important for the synthesis of four-coordinate organoboron species to ensure a completely efficient assembly of multi-component structures in a single operation [7]. Pyrazolopyridines are among the most studied condensed pyrazole systems in medicinal chemistry. E. Arbačiauskienė and coworkers reported the synthesis of a library of 2,4,6,7-tetrasubstituted-2*H*-pyrazolo[4,3-*c*]pyridines and the evaluation of their anti-proliferative activity against K562, MV4-11, and MCF-7 cancer cell lines. Out of the tested compounds, 4-(2,6-diphenyl-2*H*-pyrazolo[4,3-*c*]pyridin-7-yl)phenol proved to be the most active. Further experiments carried out by these authors revealed that it blocks proliferation and induces cell death in K562 cells. They also examined and discussed the influence of an additional substituent at the 7-position on the biological and optical properties of the compounds [8]. A. Šačkus, E. Arbačiauskienė and coworkers developed a simple and efficient synthetic route for the preparation of 3a,4-dihydro-3*H*,7*H*- and 4*H*,7*H*-pyrazolo[4′,3′:5,6]pyrano[4,3-*c*][1,2]oxazoles from easily obtainable 3-(prop-2-en-1-yloxy)- or 3-(prop-2-yn-1-yloxy)-1*H*-pyrazole-4-carbaldehydes via the intramolecular nitrile oxide cycloaddition (INOC) reaction of intermediate oximes. The key step is the nitrile oxide preparation from the corresponding aldoximes, which was carried out via oxidation with sodium hypochlorite. Pyrazolo[4′,3′:5,6]pyrano[4,3-*c*][1,2]oxazoles with various substituents at C-3 or C-7 were synthetized. Through extensive NMR spectroscopic studies carried out using standard and advanced methods, the authors unambiguously confirmed the predomination of the *syn*-isomer of intermediate aldoximes [9]. Bearing in mind the remarkable biological activities of both pyrazole and thiazole scaffolds, Y. N. Mabkhot and coworkers developed a convenient synthesis of novel pyrazolo[5,1-*b*]thiazole-based heterocycles. They also selected some examples of these compounds which were screened and evaluated for their anti-microbial and anti-cancer activities. Due to the promising results found, these compounds can be regarded as leading compounds in the future design of new drug molecules [10]. *N*-unsubstituted pyrazoles are considered versatile ligands in various fields, such as materials chemistry and homogeneous catalysis, owing to their proton-responsive nature. S. Kuwata and coworker provided an overview of the reactivities of protic pyrazole complexes. The coordination chemistry of pincer-type 2,6-bis(1*H*-pyrazol-3-yl)pyridines is first reviewed as a class of compounds for which significant advances have been made in the last decade. They described the stoichiometric reactivities of protic pyrazole complexes with inorganic nitrogenous compounds, which possibly relates to the inorganic nitrogen cycle in nature. They also outlined the catalytic application of protic pyrazole complexes, emphasizing mechanistic aspects. Moreover, they discussed the role of the NH group in the protic pyrazole ligand and the resulting metal–ligand cooperation [11]. The recent developments of multicomponent reactions for the synthesis of biologically active molecules containing the pyrazole moiety were reviewed by J.-C. Castillo and coworkers. They analyzed the articles published from 2015 to date related to anti-bacterial, anti-cancer, anti-fungal, anti-oxidant, anti-inflammatory, anti-mycobacterial, anti-malarial, α-glucosidase and α-amylase inhibitory activity, and miscellaneous activities of pyrazole derivatives obtained exclusively via a multicomponent reaction. Various plausible synthetic mechanisms and molecular docking studies were presented and discussed. This work is an important contribution for the development of more biologically active molecules and marketed drugs containing the pyrazole moiety using one-pot, atom economic synthetic strategies [12]. Vinylpyrazoles (pyrazolyl olefins) are interesting motifs in organic chemistry but have been overlooked. A. Silva and V. Silva revisited the chemistry of this type of pyrazoles. They described the properties and synthetic routes of vinylpyrazoles and highlighted their versatility as building blocks for the construction of more complex organic molecules via different organic reactions. They also gave some prospects regarding the future development of the chemistry of these interesting compounds [13]. A. Danel and coworkers summarized a little over 100 years (1911–2021) of research on the synthesis and the photophysical and biological properties of 1*H*-pyrazolo[3,4-*b*]quinolines. They reported on the main methods of synthesis, which include Friedländer condensation, synthesis from anthranilic acid derivatives, multicomponent synthesis, and others, and the use of this class of compounds as potential fluorescent sensors and biologically active compounds. This work can serve as a kind of guide for researchers who are involved in the synthesis of nitrogen-condensed heterocycles [14]. J. I. Borrell and coworkers analyzed the diversity of substituents present in the important group of heterocyclic compounds, the pyrazolo[3,4-*b*]pyridines, at N-1, C-3, C-4, C-5, and C-6. They also reported synthetic methods used for their synthesis, starting from a pre-formed pyrazole or pyridine, as well as the biomedical applications of such compounds. They summarized the most important biological targets and molecules developed, showing the high versatility of these structures [15].

In conclusion, pyrazoles’ chemistry is undoubtedly a current and relevant topic of investigation. Several research groups have contributed to the advancement of this topic by designing novel compounds and developing synthetic strategies for the preparation and post-functionalization of pyrazoles, or by studying their structures, properties, and potential applications. Herein, important advances in pyrazoles’ chemistry have been revealed. We thank all the authors for their valuable contributions to this Special Issue and offer them our immense gratitude for having chosen this Special Issue in which to publish their research works, thus placing this Special Issue in the top-ten Special Issues that collected the most papers in 2022. We are also deeply grateful to the staff members of MDPI for their editorial support.
